# First-Principles Study on the Half-Metallicity of New MXene Materials Nd_2_NT_2_ (T = OH, O, S, F, Cl, and Br)

**DOI:** 10.3389/fchem.2021.832449

**Published:** 2022-02-10

**Authors:** Kun Yang, Shuning Ren, Haishen Huang, Bo Wu, Guangxian Shen, Tingyan Zhou, Xiaoying Liu

**Affiliations:** ^1^ School of Physics and Electronic Science, Zunyi Normal University, Zunyi, China; ^2^ School of Physics and Electronic Science, Guizhou Normal University, Guiyang, China; ^3^ College of Teacher Education, Hainan Normal University, Haikou, China

**Keywords:** two-dimensional, MXene, Lanthanum series, half-metallic characteristics, spin polarization

## Abstract

This work systematically studied the structure, magnetic and electronic properties of the MXene materials Nd_2_N and Nd_2_NT_2_ (T = OH, O, S, F, Cl, and Br) via first-principles calculations based on density functional theory. Results showed that Nd_2_NT_2_ (T = OH, O, S, F, Cl, and Br) have half-metallic characteristics whose half-metallic band gap width is higher than 1.70 eV. Its working function ranges from 1.83 to 6.50 eV. The effects of strain on its magnetic and electronic structures were evaluated. Results showed that the structure of Nd_2_NT_2_ (T = OH, O, S, and Br) transitions from a ferromagnetic half-metallic semiconductor to a ferromagnetic metallic and ferromagnetic semiconductor under different strains. By contrast, the structures of Nd_2_NF_2_ and Nd_2_NS_2_ were observed to transition from a half-metallic semiconductor to a ferromagnetic metallic semiconductor under different strains. Calculations of the electronic properties of different proportions of the surface functional groups of Nd_2_NT_*x*_ (T = OH, O, and F; *x* = 0.5, 1(I, II), and 1.5) revealed that Nd_2_NO_1.5_ has the characteristics of semiconductors, whereas Nd_2_NO(II) possesses the characteristics of half-metallic semiconductors. The other structures were observed to exhibit the characteristics of metallic semiconductors. Prediction of Nd_2_NT_2_ (T = OH, O, S, F, Cl, and Br) increases the types of lanthanide MXene materials. They are appropriate candidate materials for preparing spintronic devices.

## 1 Introduction

As candidate materials for preparing spintronic devices with a high-density, a high read/write speed, and an ultra-small volume, two-dimensional ferromagnetic half-metallic materials are the key to the development of spintronic devices (Wolf et al., 2001; Hu et al., 2014; Kent and Worledge, 2015; Wang et al., 2021a). Ferromagnetic half-metallic materials have 100% spin polarization. In a spin state, they have metallic properties at the Fermi level. In another spin state, they exhibit semiconductor or insulator properties at the Fermi level. Since 2004, graphene has been experimentally prepared successfully (Novoselov et al., 2004). Graphene is a semiconductor with a zero band gap, a feature limits its application in magnetic equipment (Novoselov et al., 2004; Geim and Novoselov, 2007). Graphene can be applied to spintronic devices by improving graphene or developing graphene-like materials. Extensive research on graphene-like materials, such as hexagonal boron nitride, silicon, phosphorus, transition-metal dichalcogenides and transition-metal carbon (nitrogen) compounds (MXenes), is being conducted (Denk et al., 1994; Sevik, 2013; Liu et al., 2014; Naguib et al., 2014; Kranthi Kumar et al., 2015). MXenes have received increased attention because they have abundant types.

Two-dimensional MXene materials have been developed using HF corrosion body phase material Ti_3_AlC_2_ to remove Al atom experimentally and obtain Ti_3_C_2_ materials with a few layers (Naguib et al., 2011). MXene materials are represented by the formula M_*n*+1_X_*n*_T_*x*_ (*n* = 1, 2, 3), where M is a transition metal, X is either C or N, and T_*x*_ is a surface functional group. MXenes are becoming popular two-dimensional materials. Theoretical and experimental studies revealed that various MXene materials have intrinsic ferromagnetic half-metallicity, such as Cr_2_C, Cr_2_NO_2_, Fe_2_NO_2_, Co_2_NO_2_, Ni_2_NT_2_ (T = O, F, OH), and Mn_2_NT_2_ (T = O, OH, F) (Si et al., 2015; Wang, 2016; Wang and Liao, 2017; Frey et al., 2018). External conditions can be applied to induce Ti_2_NO_2_, Cr_3_C_2_, and Hf_2_MnC_2_O_2_ and obtain ferromagnetic half-metals (Chen et al., 2017; Zhang and Li, 2017; Siriwardane et al., 2019). However, several MXene materials have semiconductor properties, such as Sc_2_CO_2_, Ti_2_CO_2_, and Cr_2_CT_2_ (T = F, OH, O, Cl) (Lee et al., 2014; Si et al., 2015; Zhou et al., 2016). Therefore, MXenes have rich magnetic and electronic properties that must be harnessed.

Most MXene materials have different sensitivities to surface functional groups and external conditions. Therefore, MXene functional materials can be feasibly designed by exploiting functional groups or external conditions. However, current research on MXene materials mostly focuses on transition metals and largely ignores MXene materials with lanthanide elements. Tan et al. studied the strain piezoelectric coefficient of La_2_CO_2_, a lanthanide MXene material, under axial strain. They reported that strain piezoelectric coefficient of this material is up to 22.32 pm/V, which is substantially higher than that of other known piezoelectric materials, such as Sc_2_CO_2_, Y_2_CO_2_, BN, GaAs, and AlSb (Chen et al., 2021; Wang et al., 2021b). Bai et al. reported that the semiconductor MXene material Lu_2_CT_2_ (T = F, OH) has a low work function and a carrier mobility of about 105 cm^2^/V at room temperature (Zhang et al., 2021). Therefore, lanthanide MXene materials have excellent properties with great application potential in sensors, electromagnetic interference and catalysis.

M_2_N (M = Cr, Mo, W), which belongs to the VIB group in MXene materials (Hou et al., 2021), has excellent properties whose surface functional groups can induce Cr_2_NO_2_ to exhibit stable half-metallicity (Wang, 2016). On the basis of the semimetal properties of VIB MXene materials reported thus far, this work explored the structural and electromagnetic properties of the lanthanide MXene material Nd_2_NT_2_ (T = OH, O, S, F, Cl, and Br) to increase the known types of lanthanide MXene materials. This study provides theoretical guidance and direction to the preparation of related spintronic devices.

## 2 Calculation Method

First-principles calculations based on density functional theory were conducted using the CASTEP calculation package (Lin and Wang, 2017). Perdew–Burke–Ernzerhof exchange correlation function under the generalized gradient approximation was applied and ultrasoft pseudopotentials were selected to describe the interaction between electrons and ions in a two-dimensional system (Tan et al., 2019; Bai et al., 2020). In the process of geometric optimization of the structure of Nd_2_NT_2_ (T = OH, O, S, F, Cl, and Br), the two-dimensional structure preliminarily assumed was a ferromagnetic structure, and spin polarization calculations were performed. After testing the preliminary calculation parameters, the truncation energy chosen was 420 eV, the self-consistent convergence standard was set to 1 × 10^−6^ eV/atom, the sampling at *k* point was 10 × 10 × 1, and the total energy convergence standard was set to 1 × 10^−6^ eV/atom. When the atomic structure was optimized, the force of each atom was not over 0.03 eV/Å, the maximum displacement of each atom was set to 0.001 Å, and the vacuum layer of the *c* axis was set to 20 Å.

The formation energy was calculated to describe the thermodynamic stability of two-dimensional MXene material systems with different surface functional groups by using the following formula (Bekaert et al., 2020):

$$E_{\text{F}}=E_{\text{total}}\left({Nd}_{2}{NT}_{2}\right)-E_{\text{total}}\left({Nd}_{2}N\right)-E_{\text{total}}\left(T_{2}\right)$$ (1)

where *E*_total_(Nd_2_NT_2_) is the total energy of Nd_2_NT_2_, *E*_total_(Nd_2_N) is the total energy of Nd_2_N, and *E*_total_(T_2_) is the total energy of T_2_ (T = OH, O, S, F, Cl, and Br) of functional groups.

## 3 Results and Discussion

### 3.1 Surface Functional Groups of the Structure

**FIGURE 1:**
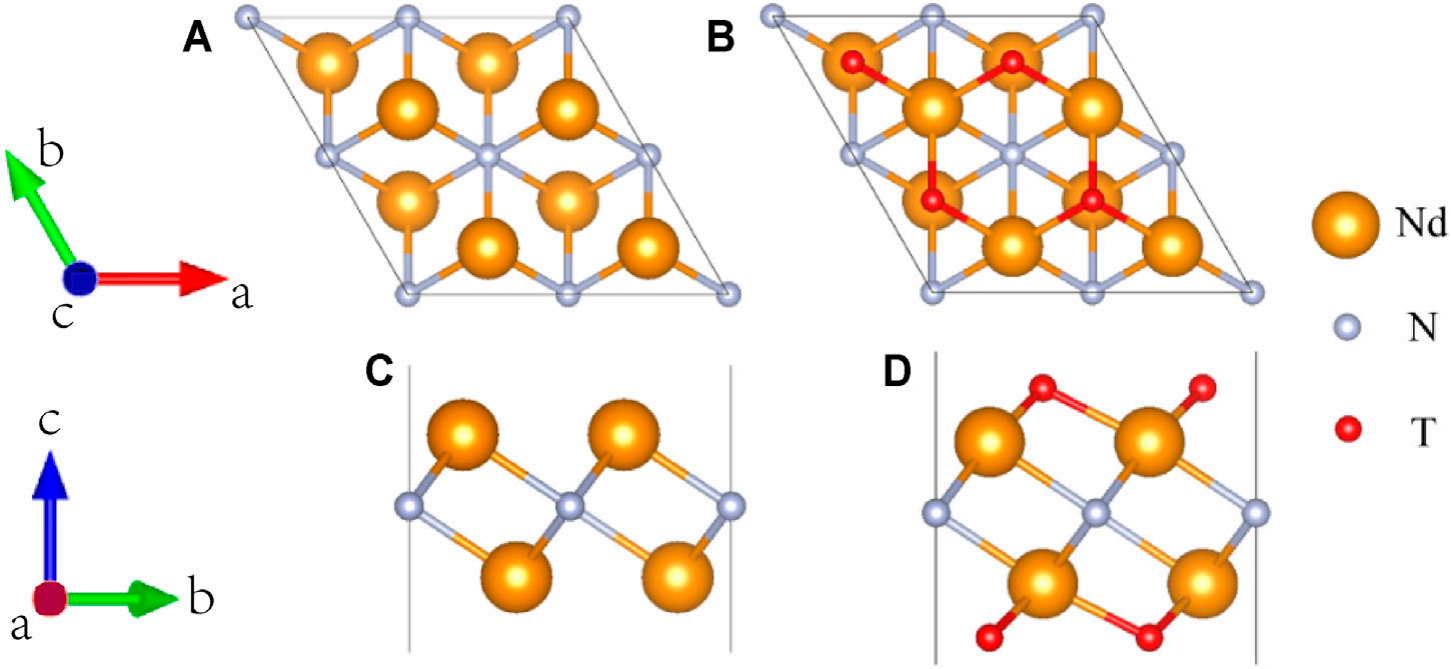
Diagram of the structure of the 2 × 2×1 supercell of the two-dimensional MXene materials **(A,C)** Nd_2_N and **(B,D)** Nd_2_NT_2_ (T = OH, O, S, F, Cl, and Br). **(A,B)** top views; **(C,D)** side views.

The top and side views of Nd_2_N after structural relaxation are shown in Figures 1A, C, respectively. The optimization results showed that Nd_2_N is a hexagonal crystal structure composed of Nd atoms on both sides and N atoms in the middle. This structure is similar to that of materials reported in the literature (Si et al., 2015; Zhang et al., 2021). Different methods for preparing MXene materials inevitably result in the formation of certain functional groups on their surface. In this study, six different functional groups present T_2_ (T = OH, O, S, F, Cl, and Br) in the optimized structure of Nd_2_N were investigated (Figures 1B, D). According to previous studies, the functional groups on the surface may be found at three sites, namely, on the top of Nd, on the top of N, and on the top of Nd at the bottom of both sides. In this study, the functional groups were observed to be located on the top of Nd at the bottom of both sides, similar to that reported in the literature (Wang and Liao, 2017; Zhang et al., 2021).

The lattice constants of Nd_2_N and Nd_2_NT_2_ and the bond lengths of Nd–N and Nd–T are listed in Table 1. The lattice constant of Nd_2_NT_2_ and the bond length of Nd–N are larger than those of Nd_2_N, indicating that the functional groups on the surface can change the structure of Nd_2_N. Nd_2_NS_2_ has the largest lattice constant (4.232 Å), whereas Nd_2_NO_2_ has the smallest lattice constant (3.755 Å). Moreover, the Nd–N and Nd–T bond lengths of Nd_2_NS_2_ are longer than those of Nd_2_NO_2_ because both O and S belong to the VIA family and have similar properties. The radius of the S atom is greater than that of the O atom. The lattice constants of Nd_2_NF_2_, Nd_2_NCl_2_, and Nd_2_NBr_2_ are 3.796, 3.911, and 3.971 Å, respectively. The lattice constants increase with the increase in the atomic radius of the VIIA family, and the bond lengths of Nd–N and Nd–T gradually increase. However, the bond length of Nd–T is quite different. Nd–Br has the maximum bond length (3.015 Å), whereas Nd–O has the minimum bond length (2.280 Å), indicating that the bonding intensity of Nd and O is greater than that of Nd–Br. However, the bond length of Nd–N is 2.62–2.69 Å, indicating that although the different surface functional groups have an effect on the structure of the monolayer Nd_2_N, their effect on the bond length of this structure is less than that on its lattice constant.

**TABLE 1:** Lattice constants of the two-dimensional MXene materials Nd_2_N and Nd_2_NT_2_ and bond lengths of Nd–N and Nd–T (T = OH, O, S, F, Cl, and Br).

Type	Nd_2_N	Nd_2_N(OH)_2_	Nd_2_NO_2_	Nd_2_NS_2_	Nd_2_NF_2_	Nd_2_NCl_2_	Nd_2_NBr_2_
*a* = *b*/Å	3.743	3.785	3.755	4.232	3.796	3.911	3.971
*d*_Nd-N_/Å	2.595	2.630	2.670	2.786	2.633	2.667	2.686
*d*_Nd-N_/Å	—	2.541	2.280	2.738	2.457	2.857	3.015

### 3.2 Magnetic and Thermodynamic Stability

The atomic resolution magnetic moments and formation energies of Nd_2_N and Nd_2_NT_2_ (T = OH, O, S, F, Cl, and Br) were calculated to assess their magnetic and thermodynamic stability (Table 2). The magnetic moments of monolayer Nd_2_N (up to 8.95 *μ*_B_) are higher than those of Nd_2_NT_2_ (T = OH, O, S, F, Cl, and Br). However, the magnetic moments of Nd_2_N(OH)_2_, Nd_2_NF_2_, Nd_2_NCl_2_, Nd_2_NBr_2_, Nd_2_NO_2_, and Nd_2_NS_2_ are 7.01, 7.01, 7.00, 7.00, 5.00, and 4.98 *μ*_B_, respectively. The magnetic moment of Nd_2_N mainly comes from two Nd atoms, and the contribution of the N atom to the total magnetic moment is small. The Nd atom is still the main contributor to the magnetic moment of Nd_2_N(OH)_2_. However, with the addition of –OH, the total magnetic moment is reduced to 7.01 *μ*_B_. The magnetic moment of the nonmagnetic elements is also reduced because of the strengthening of hybridization between the atoms that strengthened the magnetic coupling effect and reduced the magnetic moment. In the structures of Nd_2_NO_2_ and Nd_2_NS_2_, the total magnetic moment is substantially reduced primarily because of the fact that the magnetic moment of the N atom of the Nd atom is remarkably smaller. Moreover, the magnetic moments of the nonmagnetic elements O and S in the induced surface functional groups are considerably smaller than those of the magnetic elements, indicating that the bonds between the O and S atoms and Nd atom are relatively intense, resulting in a sharp decrease in atomic localization. In addition, the total magnetic moment of the structures of Nd_2_NF_2_, Nd_2_NCl_2_, and Nd_2_NBr_2_ remains 7.00 *μ*_B_. The increase in the amplitude of Nd and the F, Cl, and Br atoms is the same as that in the decrease in their amplitude. By comparison, the magnetic moment of the N atom remains low.

**TABLE 2:** Total atomic magnetic moment (*M*_total_) and atomic resolution magnetic moment (*M*), and formation energy (*E*_F_) of the two-dimensional MXene materials Nd_2_N and Nd_2_NT_2_ (T = OH, O, S, F, Cl, and Br).

Structure	*M*_Nd_(*μ*_B_)	*M*_N_(*μ*_B_)	*M*_T_(*μ*_B_)	*M*_total_(*μ*_B_)	*E*_F_(eV)
Nd_2_N	9.04	–0.09	—	8.95	—
Nd_2_N(OH)_2_	7.24	–0.19	–0.04	7.01	–25.92
Nd_2_NO_2_	6.46	–0.88	–0.58	5.00	–16.98
Nd_2_NS_2_	6.76	–1.02	–0.76	4.98	–10.42
Nd_2_NF_2_	7.24	–0.19	–0.04	7.01	–14.75
Nd_2_NCl_2_	7.26	–0.20	–0.06	7.00	–10.47
Nd_2_NBr_2_	7.28	–0.20	–0.08	7.00	–9.016

**FIGURE 2:**
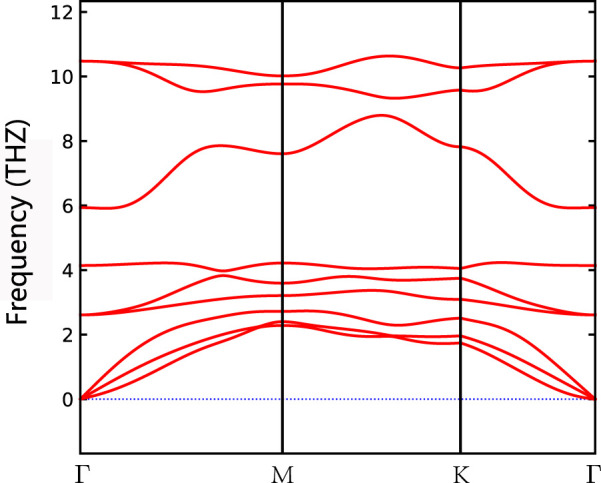
Phonon spectrum of Nd_2_N structure.

After the structural and magnetic properties of the materials were determined, the phonon spectrum of monolayer Nd_2_N and the formation energy of Nd_2_NT_2_ (T = OH, O, S, F, Cl, and Br) were further calculated. According to the phonon spectrum of Nd_2_N, it has good dynamic stability (Figure 2). Equation 1 was also used to calculate the formation energy of Nd_2_NT_2_ (T = OH, O, S, F, Cl, and Br). Nd_2_N(OH)_2_ has the smallest formation energy of –25.92 eV, whereas Nd_2_NBr_2_ has the largest formation energy of –9.016 eV (Table 2), indicating an intense interaction between the metals and the surface functional groups. When the surface functional groups are F, Cl, and Br (VIIA group), the formation energy gradually decreases (Table 2). However, the surface functional groups O and S (VIA group) also show a similar rule. According to previous studies, on the surface functional groups of MXene structures, the formation energy can easily change from high to low under certain conditions. The formation energy of the –OH structure with surface functional groups is smaller than that of the O, S, F, Cl, and Br structures with surface functional groups (Table 2), suggesting that MXene materials with O, S, F, Cl, and Br as functional groups should not be washed or stored in H_2_O during preparation to prevent them from being converted into –OH MXene materials (Perdew et al., 1996).

### 3.3 Electronic Properties

**FIGURE 3:**
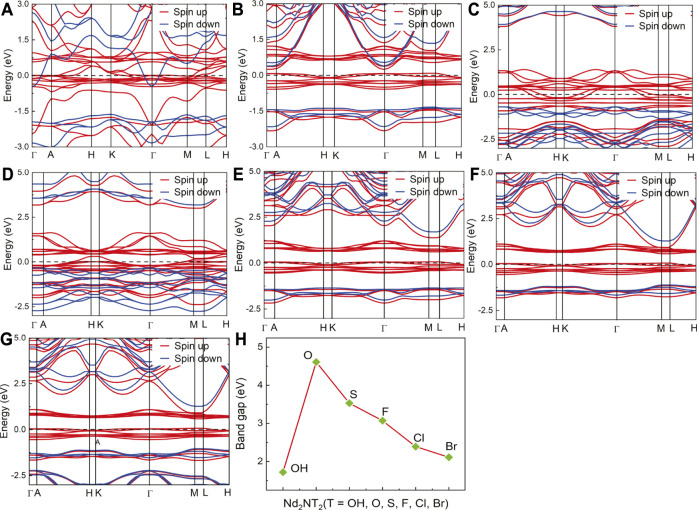
Energy band structure and band gap width diagram of the two–dimensional MXene materials Nd_2_N and Nd_2_NT_2_ (T = OH, O, S, F, Cl, and Br). **(A)** Nd_2_N, **(B)** Nd_2_N(OH)_2_, **(C)** Nd_2_NO_2_, **(D)** Nd_2_NS_2_, **(E)** Nd_2_NF_2_, **(F)** Nd_2_NCl_2_, **(G)** Nd_2_NBr_2_, and **(H)** band gap width diagram.

The electronic properties of Nd_2_N and Nd_2_NT_2_ (T = OH, O, S, F, Cl, and Br) were evaluated by calculating and plotting the energy band structure diagram (Figure 3). The spin up and the spin down of the energy band structure of Nd_2_N pass through the Fermi level, indicating that it has ferromagnetic characteristics. In the spin down channel, the energy band density near the Fermi level is relatively sparse, indicating that the energy band structure can be changed under certain conditions, which are described below.

1) In the energy band structure of Nd_2_NT_2_ (T = OH, F, Cl, and Br), the spin up energy band at the Fermi level passes through the Fermi level, whereas the spin down energy band has an energy band gap, indicating that it has semimetal characteristics. The spin up energy band has an energy band gap near the Fermi level, indicating that the structure may change from a semimetal to a semiconductor under certain conditions.
2) In the band structure of Nd_2_NT_2_ (T = O and S), the spin up band at the Fermi level passes through the Fermi level, suggesting that it has metal characteristics. However, the spin down band has an energy band gap at the Fermi level, reflecting the nature of a semiconductor. Therefore, there is 100% spin polarization at the Fermi level, indicating that it has semimetal characteristics. According to the energy band structure, the band gap width between the spin down valence band and the Fermi surface is approximately 0.2–0.6 eV, and the band gap width between the spin down conduction band and the Fermi surface is greater than 3 eV, indicating that the structure does not readily change from a half-metal to a metal. Theoretically, the half-metallicity remains stable under certain external conditions.
3) The spin down band gap width of the semimetal was counted. Nd_2_N(OH)_2_ has the smallest band gap width (1.72 eV), whereas Nd_2_NO_2_ has the largest band gap width (4.61 eV) (Figure 3H). Therefore, the half-metallicity can remain stable within a certain range.

**FIGURE 4:**
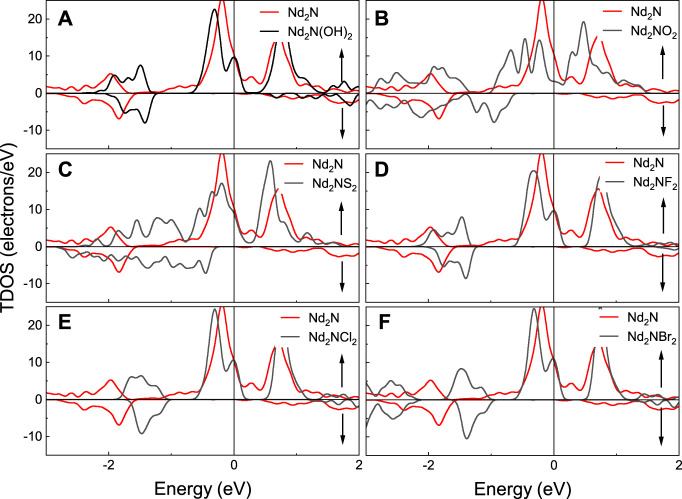
Total density of states (TDOS) of Nd_2_N and Nd_2_NT_2_ (T = OH, O, S, F, Cl, and Br). The red line represents Nd_2_N, whereas the black line denotes is **(A)** Nd_2_N(OH)_2_, **(B)** Nd_2_NO_2_, **(C)** Nd_2_NS_2_, **(D)** Nd_2_NF_2_, **(E)** Nd_2_NCl_2_, **(F)** Nd_2_NBr_2_, respectively.

The calculated total density of states (TDOS) of Nd_2_N and Nd_2_NT_2_ (T = OH, O, S, F, Cl, and Br) are provided in Figure 4. As can be seen from the TDOS graphs, the polarization peak of Nd_2_N(OH)_2_ appears at 1.5 eV, but this phenomenon is not observed in Nd_2_N. Moreover, the spin down band gaps of Nd_2_NO_2_ and Nd_2_NS_2_ move to the high energy region, and their band gap width increase. However, the spin down band gaps of Nd_2_NF_2_, Nd_2_NCl_2_, and Nd_2_NBr_2_ exhibit similar behavioral changes, and the only differences are in the energy range of spin polarization peaks, which gradually move to the high-energy region.

### 3.4 Work Functions of Nd_2_NT_2_ (T = OH, O, S, F, Cl, and Br)

**FIGURE 5:**
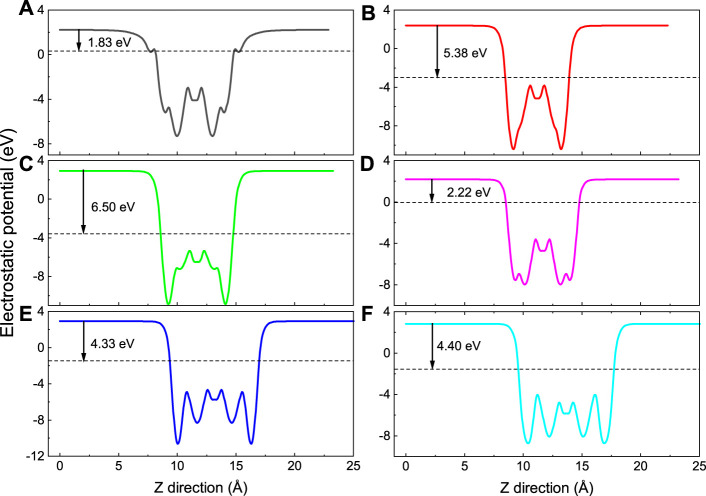
Calculated electrostatic potentials along the *Z* direction of Nd_2_NT_2_ (T = OH, O, S, F, Cl, and Br), while **(A)** Nd_2_N(OH)_2_, **(B)** Nd_2_NO_2_, **(C)** Nd_2_NS_2_, **(D)** Nd_2_NF_2_, **(E)** Nd_2_NCl_2_, **(F)** Nd_2_NBr_2_, respectively. The black arrow indicates the difference from vacuum level to the Fermi level. The black dashed line denotes the Fermi level.

Work function, as a reference for charge transfer, is a key parameter. The definition of work function (*φ*_wf_) is as follows (Vanderbilt, 1990):

$$\varphi_{\text{wf}}=E_{\text{vacuum}}-E_{\text{F}}$$ (2)

where *E*_vacuum_ represents the energy of the surface electron energy level in the vacuum, and *E*_F_ is the Fermi level of the MXene material. The electrostatic potentials of Nd_2_NT_2_ (T = OH, O, S, F, Cl, and Br) are calculated using Eq. 2 and plotted in Figure 5. The work function of Nd_2_N is approximately 1.186 eV, whereas that of Nd_2_N(OH)_2_, Nd_2_NO_2_, Nd_2_NS_2_, Nd_2_NF_2_, Nd_2_NCl_2_, and Nd_2_NBr_2_ is 1.83, 5.38, 6.50, 2.22, 4.33, and 4.40 eV, respectively. The work function of –OH is 1.6–2.8 eV, similar to that reported in the literature (Khazaei et al., 2013). Moreover, the work function of Nd_2_NT_2_ (T = OH, F, Cl, and Br) increases as VIIA atoms are introduced. The work function of Nd_2_NO_2_ is evidently lower than that of Nd_2_NS_2_. The work functions indicate that lanthanide MXene materials have potential applications in spintronic devices.

### 3.5 Strain Effect

**FIGURE 6:**
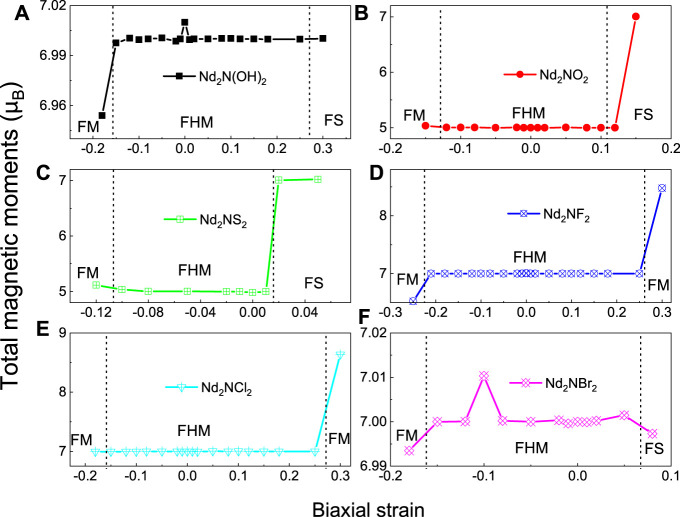
Total magnetic moments of monolayer Nd_2_NT_2_ (T = OH, O, S, F, Cl, and Br) under biaxial strain. **(A)** Nd_2_N(OH)_2_, **(B)** Nd_2_NO_2_, **(C)** Nd_2_NS_2_, **(D)** Nd_2_NF_2_, **(E)** Nd_2_NCl_2_, **(F)** Nd_2_NBr_2_. FM, FHM, and FS denote ferromagnetic metallic, ferromagnetic half-metallic, and ferromagnetic semiconductor, respectively.

The effects of strain on the magnetic and electronic structure of monolayer Nd_2_NT_2_ (T = OH, O, S, F, Cl, and Br) were evaluated by calculating the magnetic moment under different biaxial strains (Figure 6). Theoretical studies indicated that the electronic and magnetic properties of the monolayer MXenes Ti_2_C and Ti_2_N are tunable by strain (Clark et al., 2005; Sternik and Wdowik, 2018). Thus, biaxial strain was applied to monolayer Nd_2_NT_2_ (T = OH, O, S, F, Cl, and Br) by using the following formula:

$$\varepsilon =\frac{L-L_{0}}{L_{0}}$$ (3)

where *L* and *L*_0_ are the lattice constants of strain and the equilibrium, respectively. Positive and negative *ɛ* values correspond to tensile and compressive strain, respectively. Under different strains, both Nd_2_NF_2_ and Nd_2_NCl_2_ transition from a ferromagnetic half-metallic structure to a ferromagnetic metallic structure (Figure 6), and their total magnetic moment is maintained is from 6.5 to 9.0 *μ*_B_. By comparison, Nd_2_N(OH)_2_, Nd_2_NO_2_, Nd_2_NS_2_, and Nd_2_NBr_2_ transition from a ferromagnetic half-metallic structure to a ferromagnetic metallic structure and a ferromagnetic semiconductor under different strains. Notably, the total magnetic moments of the transition of Nd_2_NO_2_ and Nd_2_NS_2_ range from 5 to 7 *μ*_B_under strain.

### 3.6 Electronic Properties of Different Proportions of the Surface Functional Groups of Nd_2_NT_*x*_ (T = OH, O, F; x = 0.5, 1(I, II), and 1.5)

**FIGURE 7:**
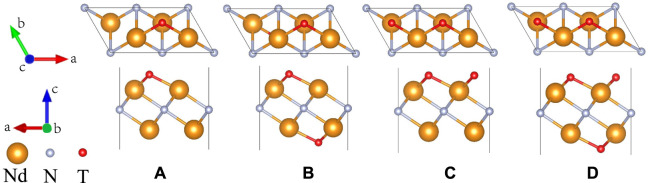
Top and side views of the 2 × 1×1 supercell of the structure of the two-dimensional MXene material Nd_2_NT_*x*_ (T = OH, O, F; *x* = 0.5, 1, and 1.5). **(A)** Nd_2_NT_0.5_ (T = OH, O, F), **(B)** Nd_2_NT (T = OH, O, F)(I), **(C)** Nd_2_NT (T = OH, O, F)(II), and **(D)** Nd_2_NT_1.5_ (T = OH, O, F).

**FIGURE 8:**
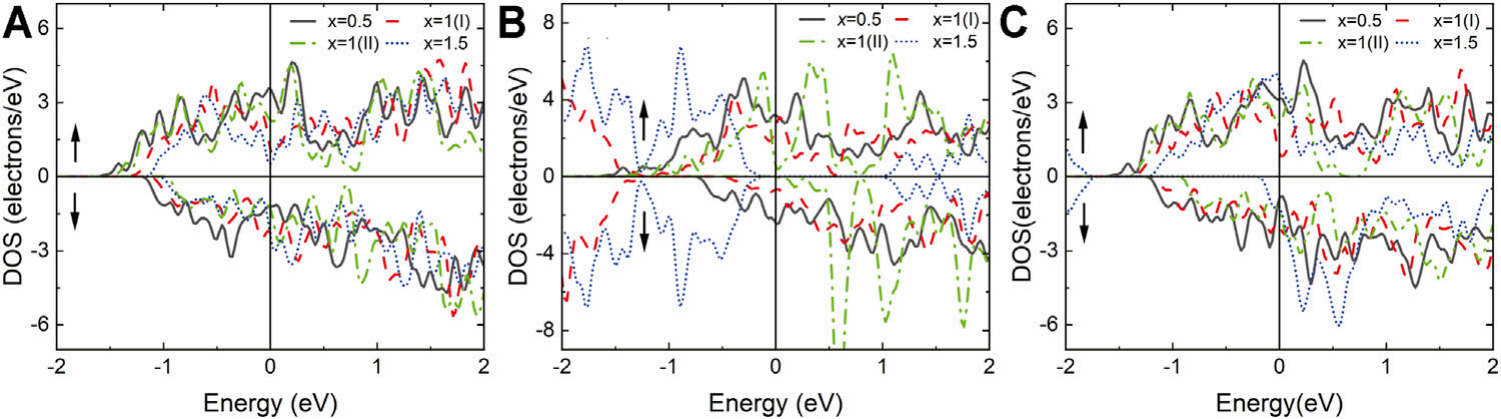
Total density of state(DOS) of **(A)** Nd_2_N(OH)_*x*_, **(B)** Nd_2_NO_*x*_, and **(C)** Nd_2_NF_*x*_, where *x* = 0.5, 1(I), 1(II), and 1.5.

Surface functional groups have a great influence on the electronic properties of MXene materials (Xie et al., 2014). The influence of different proportions of the surface functional groups of the Nd_2_NT_*x*_ materials on their electronic properties was assessed at proportions of *x* = 0.5, 1, and 1.5 (Figure 7). *x* = 1 has two types; in type I, the functional groups are distributed on both sides, whereas in type II, the functional groups are distributed on one side only. After geometry optimization, TDOS was calculated (Figure 8).

The structures of Nd_2_N(OH)_*x*_ and Nd_2_NF_*x*_ exhibit metal characteristics because of the asymmetry of their surface functional groups. When the surface functional group is 100%, the material has half-metallic characteristics. This result provides a direction for detecting the richness of surface functional groups experimentally.

When *x* = 0.5 and 1 (type I), the structure of Nd_2_NO_*x*_ exhibits metal characteristics. However, when *x* = 1 (type II), an obvious band gap is observed in the spin downward at the Fermi level, indicating that it has half-metal characteristics. Unexpectedly, when *x* = 1.5, both spin up and spin down have a symmetric band gap width at the Fermi level, indicating that it has the characteristics of a semiconductor. This result provides a reliable direction for the regulation of electronic properties by controlling the richness of surface functional groups experimentally.

## 4 Discussion

The structural, magnetic, and electronic properties of Nd_2_N and Nd_2_NT_2_ (T = OH, O, S, F, Cl, and Br) were evaluated via first-principles calculations based on density functional theory. According to the calculated phonon spectrum and formation energies, Nd_2_N and Nd_2_NT_2_ (T = OH, O, S, F, Cl, and Br) are stable. Owing to the effects of surface functional groups on the electronic properties of Nd_2_NT_2_ (T = OH, O, S, F, Cl, and Br), it exhibits the characteristics of a half-metal, and its band gap width is higher than 1.70 eV. The work function ranges from 1.83 to 6.50 eV, indicating that lanthanide MXene materials have potential applications in spintronic devices. Under different strains, Nd_2_NT_2_ (T = OH, O, S, and Br) transition from a ferromagnetic half-metallic structure to a ferromagnetic metallic structure and a ferromagnetic semiconductor. However, the structures of Nd_2_NF_2_ and Nd_2_NS_2_ transition from a half-metallic structure to a ferromagnetic metallic structure under different strains. Calculation of the electronic properties of different proportions of the surface functional groups of Nd_2_NT_*x*_ (T = OH, O, F; *x* = 0.5, 1(I, II), and 1.5) revealed that Nd_2_NO_1.5_ has the characteristics of a semiconductors, whereas Nd_2_NO(II) has the characteristics of a half-metal. The other structures show the characteristics of a metal. This study demonstrated that new lanthanide MXene materials have a high application potential in spintronic devices.

## Data Availability

The original contributions presented in the study are included in the article/Supplementary Material, further inquiries can be directed to the corresponding author.
